# Supplementary material for: The Impact of Edema on MRI Radiomics for the Prediction of Lung Metastasis in Soft Tissue Sarcoma
Source: Diagnostics (Basel). 2023 Oct 5;13(19):3134. doi: 10.3390/diagnostics13193134 (PMC10572878; doi:10.3390/diagnostics13193134)
Supplement: Supplementary file 1 [file diagnostics-13-03134-s001.zip › Supplementary Materials.pdf]

Figure S1. Curves computed for Gross Tumoral Volume (GTV) feature selection (AUC vs. incremental increase of features). The combination of two features was selected because of the first peak of AUC along the curve. More details for the selected features can be found in the Pyradiomics feature documentation[1]

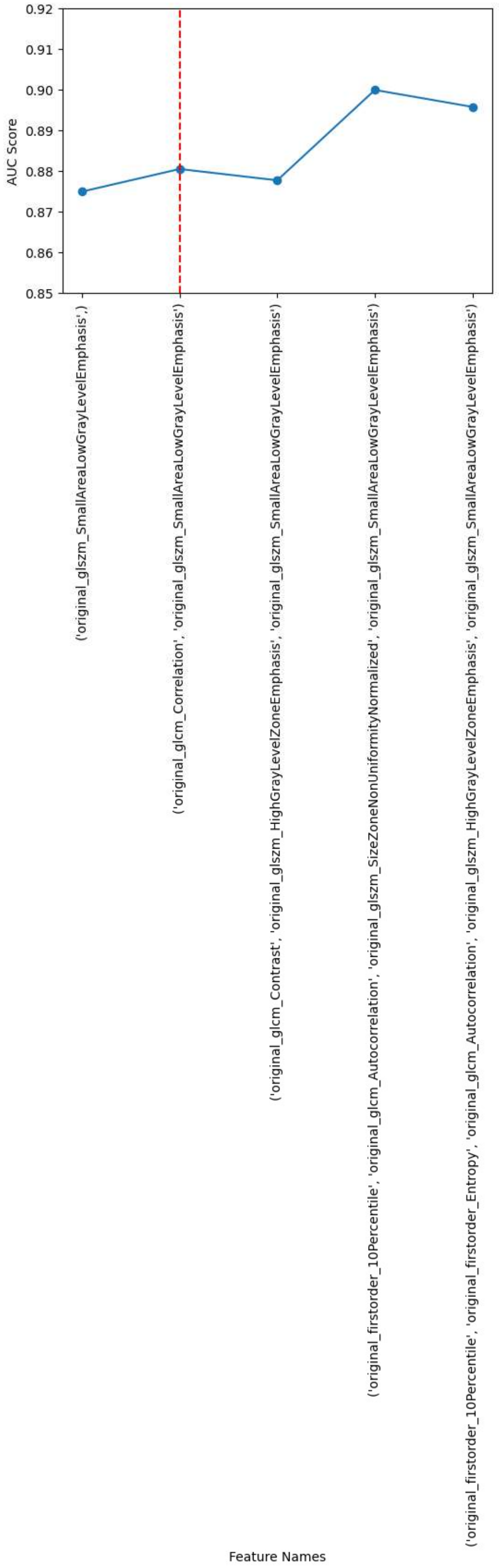

Figure S2. Curves computed for Edema Tumoral Volume (EDV) feature selection (AUC vs. incremental increase of features). The combination of two features was selected because of the first peak of AUC along the curve. More details for the selected features can be found in the Pyradiomics feature documentation[1]

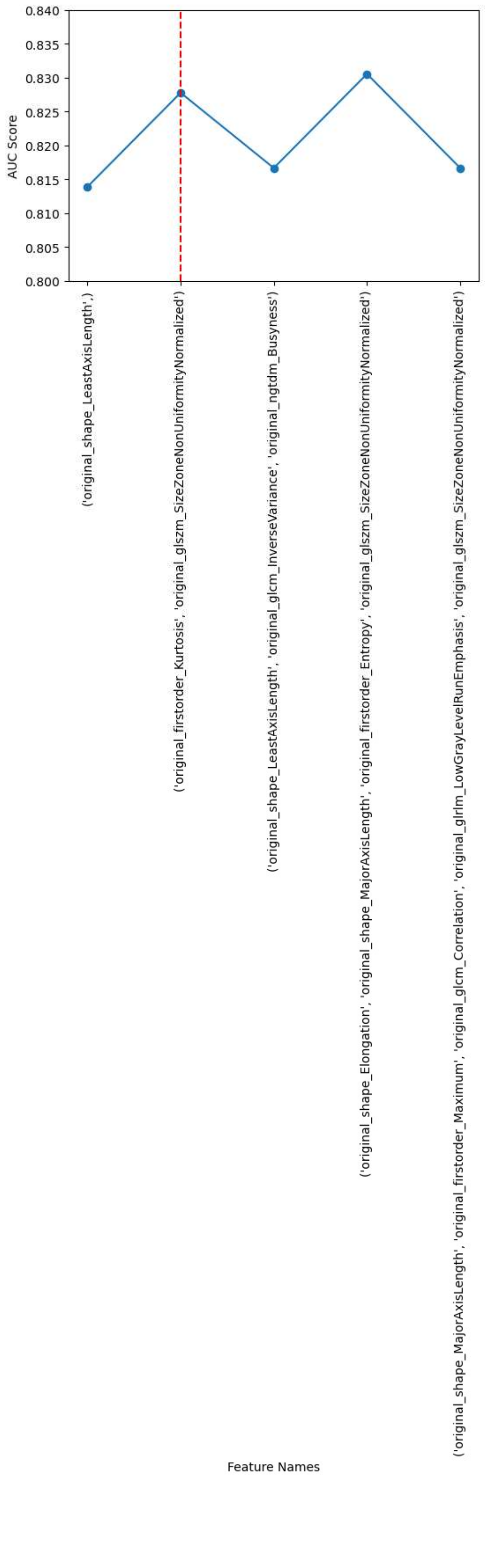

Figure S3. Histogram depicting the distribution of accuracies for the RF-GTV model (10000 bootstrap iterations[2]).

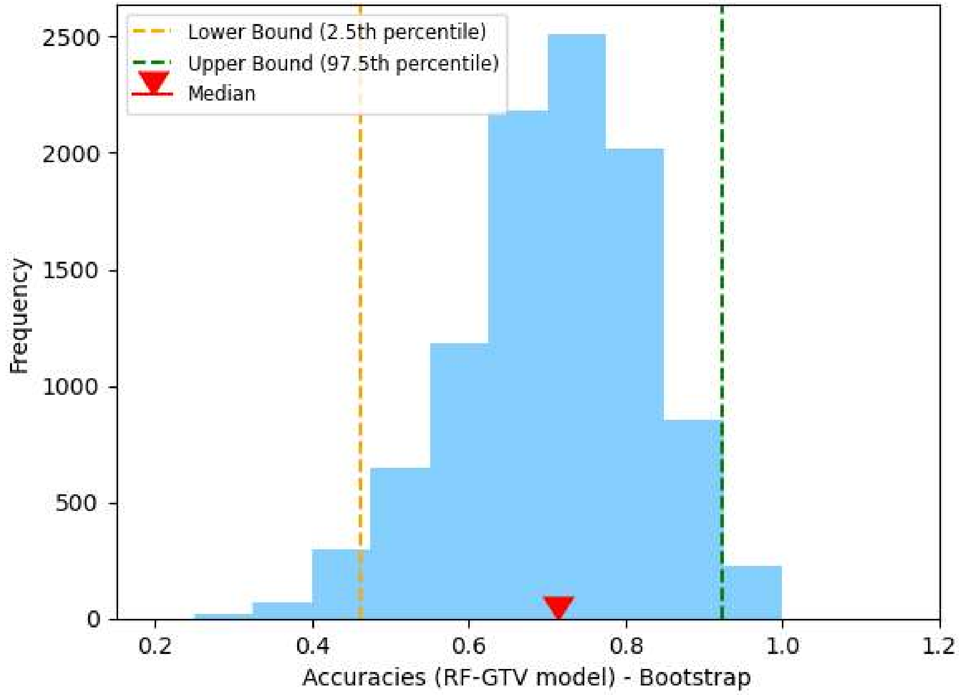

Figure S4. Histogram depicting the distribution of AUCs for the RF-GTV model (10000 bootstrap iterations[2]).

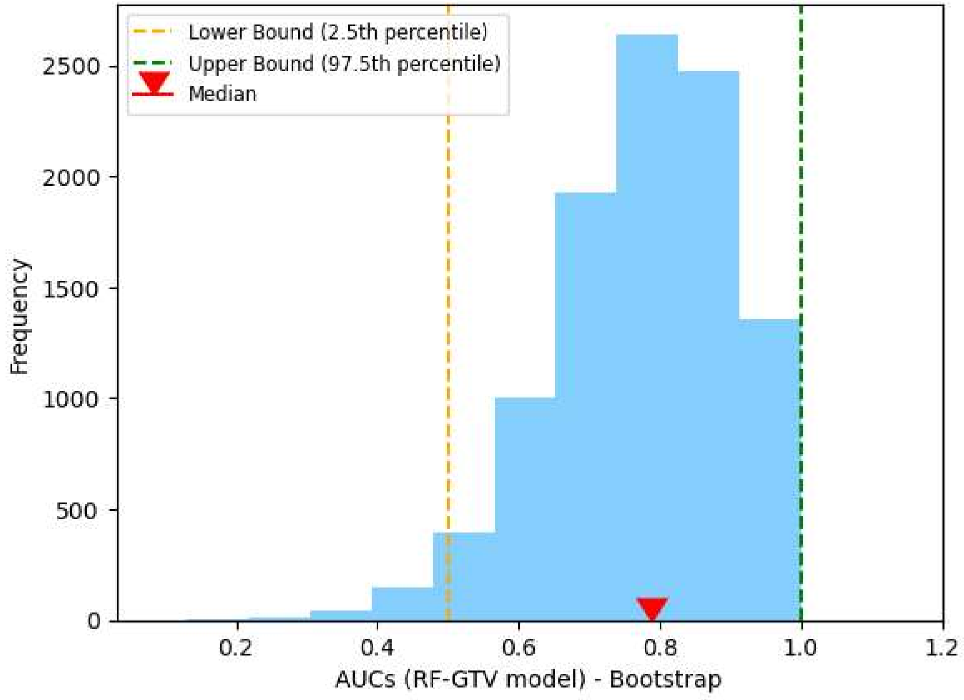

Figure S5. Histogram depicting the distribution of accuracies for the RF-EDV model (10000 bootstrap iterations[2]).

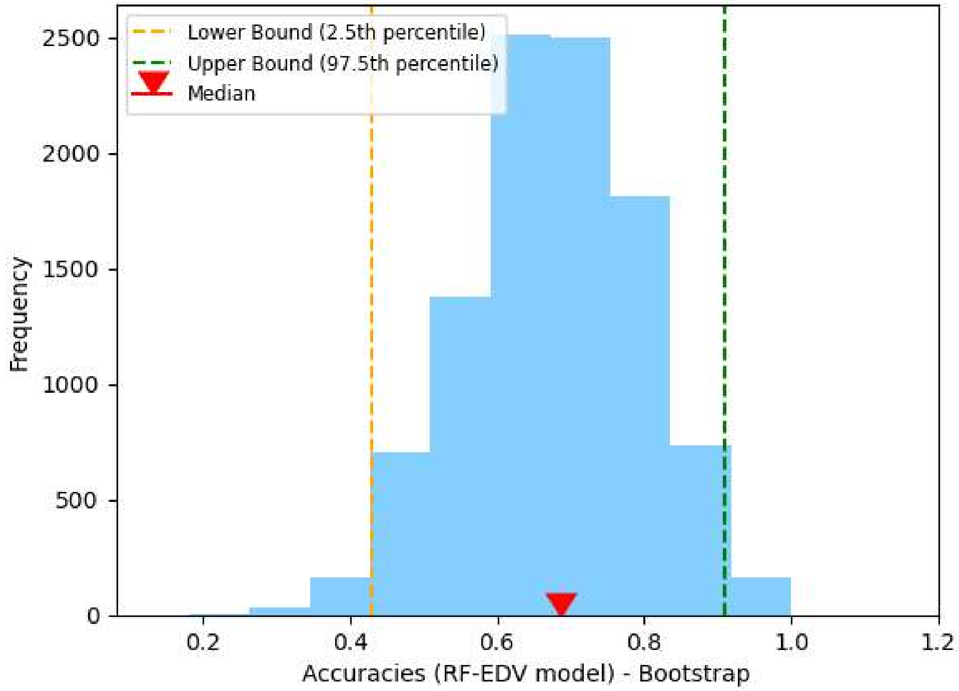

Figure S6. Histogram depicting the distribution of AUCs for the RF-EDV model (10000 bootstrap iterations[2]).

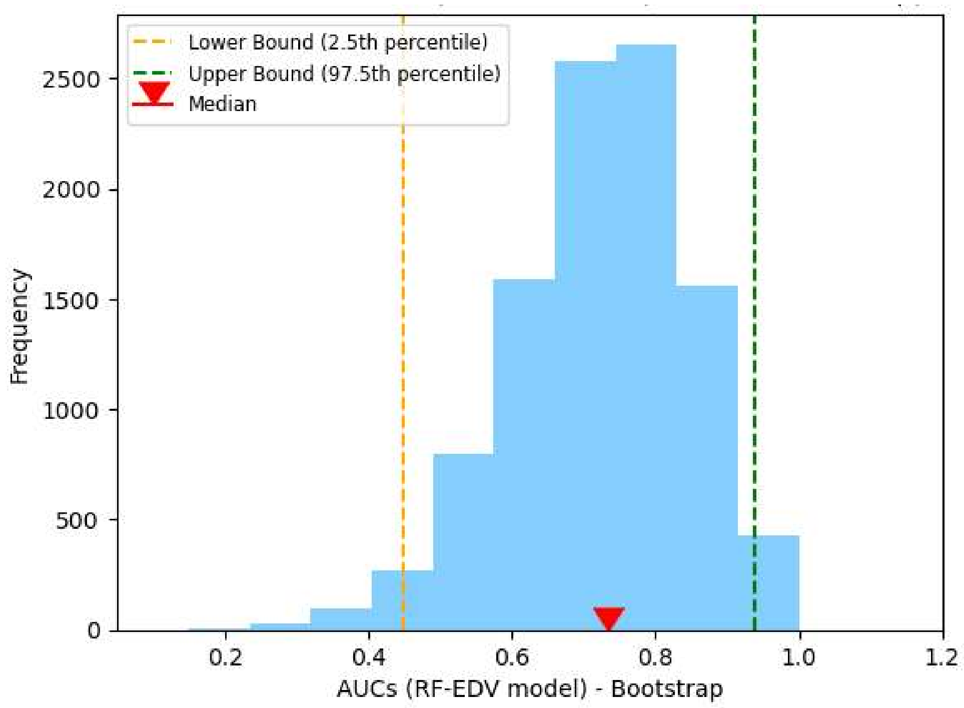

Figure S7. ROC curves, along with the corresponding AUC values, for both the RF-GTV and RF-EDV models using 10000 bootstrap iterations[2].

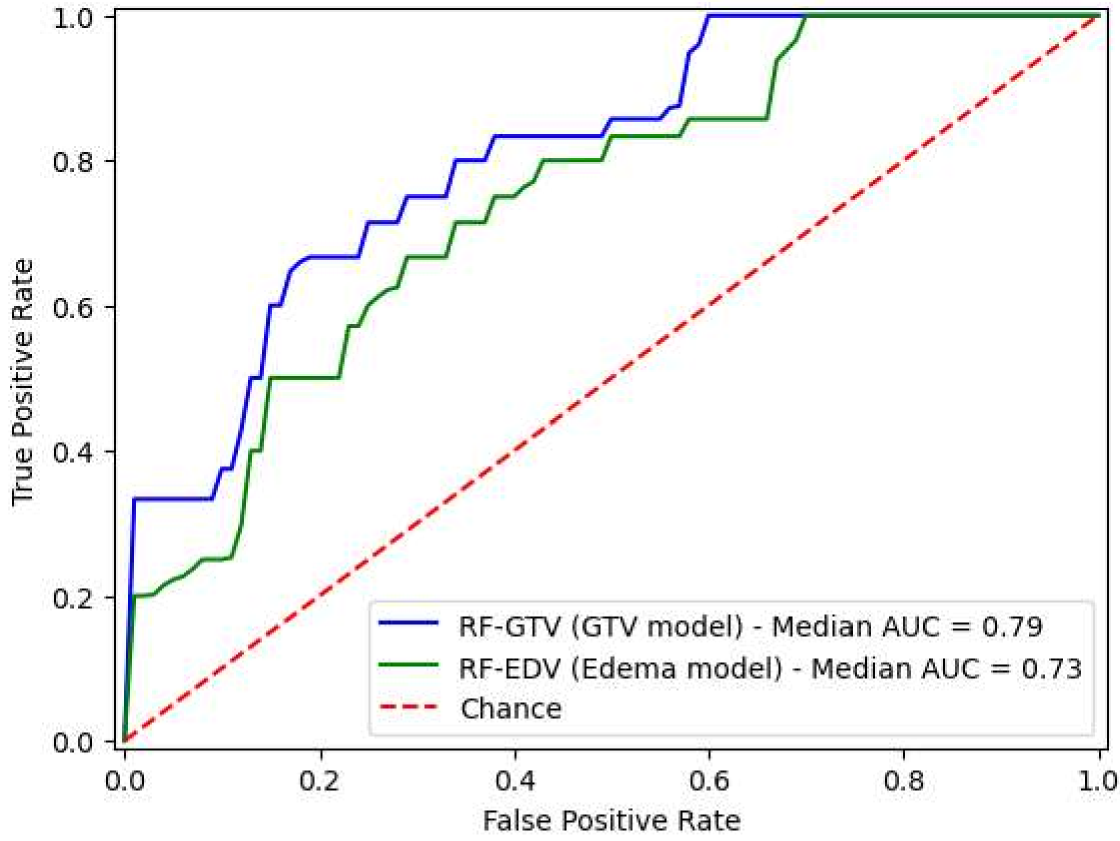

Algorithm S1: Exhaustive Feature Selection algorithm.

The Exhaustive Feature Selection algorithm employed in this study follows a wrapper approach[3] for a comprehensive evaluation of feature subsets. This method selects the optimal subset by optimizing a designated performance metric, utilizing a given regressor or classifier, and specifying both the minimum and maximum number of features to be selected as required.

For instance, when using a Random Forest (RF) classifier[4] with a dataset comprising three features (Feature_A, Feature_B, Feature_C), and specifying a Minimum and Maximum number of features to be selected as two, the algorithm systematically evaluates all possible feature combinations, which in this case include:

Feature_A, Feature_B

Feature_A, Feature_C

Feature_B, Feature_C

The algorithm then selects the combination that yields the highest performance metric, such as classification AUC value for the RF classifier.

More detail regarding the Exhaustive Feature Selection algorithm and implementation in [5].

Hardware employed and computation duration.

The data underwent processing and analysis using a MacBook Pro (Retina, 15-inch, Mid 2015) equipped with a 2.5 GHz Quad-Core Intel Core i7 processor, 16 GB of 1600 MHz DDR3 memory, and Intel Iris Pro graphics with 1536 MB of capacity.

In terms of computation duration, the feature selection process, particularly with the Exhaustive Feature Selection algorithm, consumed approximately 60 hours. The combined tasks of conducting 100 random subsampling iterations, 10,000 bootstrap iterations, and subsequent statistical computations required approximately 2.5 hours to complete.

References

1. van Griethuysen JJM, Fedorov A, Parmar C, Hosny A, Aucoin N, Narayan V, et al. Computational Radiomics System to Decode the Radiographic Phenotype. Cancer Res. 2017;77: e104–e107.

2. Efron B. Bootstrap methods: Another look at the jackknife. Ann Stat. 1979;7: 1–26.

3. Kohavi R, John GH. The wrapper approach. Feature Extraction, Construction and Selection. Boston, MA: Springer US; 1998. pp. 33–50.

4. Ho TK. Random decision forests. Proceedings of 3rd International Conference on Document Analysis and Recognition. IEEE Comput. Soc. Press; 2002. doi:10.1109/icdar.1995.598994

5. Raschka S. MLxtend: Providing machine learning and data science utilities and extensions to Python’s scientific computing stack. J Open Source Softw. 2018;3: 638.
